# Trends in HIV Terminology: Text Mining and Data Visualization Assessment of International AIDS Conference Abstracts Over 25 Years

**DOI:** 10.2196/publichealth.8552

**Published:** 2018-05-04

**Authors:** Nicole Dancy-Scott, Gale A Dutcher, Alla Keselman, Colette Hochstein, Christina Copty, Diane Ben-Senia, Sampada Rajan, Maria Guadalupe Asencio, Jason Jongwon Choi

**Affiliations:** ^1^ Division of Specialized Information Services National Library of Medicine Bethesda, MD United States; ^2^ ICF Fairfax, VA United States

**Keywords:** acquired immunodeficiency syndrome, data mining, history, HIV infections, terminology

## Abstract

**Background:**

The language encompassing health conditions can also influence behaviors that affect health outcomes. Few published quantitative studies have been conducted that evaluate HIV-related terminology changes over time. To expand this research, this study included an analysis of a dataset of abstracts presented at the International AIDS Conference (IAC) from 1989 to 2014. These abstracts reflect the global response to HIV over 25 years. Two powerful methodologies were used to evaluate the dataset: text mining to convert the unstructured information into structured data for analysis and data visualization to represent the data visually to assess trends.

**Objective:**

The purpose of this project was to evaluate the evolving use of HIV-related language in abstracts presented at the IAC from 1989 to 2014.

**Methods:**

Over 80,000 abstracts were obtained from the International AIDS Society and imported into a Microsoft SQL Server database for data processing and text mining analyses. A text mining module within the KNIME Analytics Platform, an open source software, was then used to mine the partially processed data to create a terminology corpus of key HIV terms. Subject matter experts grouped the terms into categories. Tableau, a data visualization software, was used to visualize the frequency metrics associated with the terms as line graphs and word clouds. The visualized dashboards were reviewed to discern changes in terminology use across IAC years.

**Results:**

The major findings identify trends in HIV-related terminology over 25 years. The term “AIDS epidemic” was dominantly used from 1989 to 1991 and then declined in use. In contrast, use of the term “HIV epidemic” increased through 2014. Beginning in the mid-1990s, the term “treatment experienced” appeared with increasing frequency in the abstracts. Use of terms identifying individuals as “carriers or victims” of HIV rarely appeared after 2008. Use of the terms “HIV positive” and “HIV infected” peaked in the early-1990s and then declined in use. The terms “men who have sex with men” and “MSM” were rarely used until 1994; subsequently, use of these terms increased through 2014. The term “sex worker” steadily increased in frequency throughout conference years, whereas the term “prostitute” decreased over time.

**Conclusions:**

The results of this study highlight changes in HIV terminology use over 25 years, including the addition, disappearance, and changing use of terms that reflect advances in HIV research and medical practice and destigmatization of the disease. Coupled with findings from related quantitative research, HIV-related terminology recommendations based on results of this study are included. Adoption of these recommendations will further efforts to use less stigmatizing language and facilitate effective communication between health professionals and people affected by HIV.

## Introduction

### Background

Since the first AIDS cases were reported in the United States in June 1981 [1], the vocabulary of HIV has evolved with advances in HIV research and medical practice. Text mining and visualization provide a method to investigate this evolution of HIV terminology. Using this combination strategy, the cultural and linguistic trajectory of HIV-related societal discourse can be characterized more precisely than possible with less structured approaches.

The relationship between progress to understand and treat diseases and the surrounding conversation is bidirectional. This mutual influence extends to all spheres of communication, including between patient and provider, and can influence behaviors and affect health outcomes.

For example, the term “AIDS carrier” was used often at the onset of the HIV epidemic. With the discovery of the virus that causes AIDS, the term was recognized as both biologically incorrect and inherently stigmatizing, potentially creating and reinforcing negative stereotypes of people living with HIV [2]. Kelly et al evaluated the language used to refer to people with substance use disorders. This research showed that “The language used to describe health conditions reflects and influences our attitudes and approaches to addressing them, even to the extent of suggesting that a health condition is a moral, social, or criminal issue” [3]. Stigmatizing language that assigns blame can deter individuals from seeking care and even affect clinicians’ treatment decisions. When language helps people to feel empowered, they are more likely to seek care [3].

In addition, language often reflects sociocultural and historical trends over time [4]. Michel et al created a corpus containing more than 5 million digitized books—4% of all books ever published—to quantitatively investigate cultural trends. This research demonstrated that computational analysis of digitized texts (*culturomics*) offers insights across diverse fields, including the evolution of grammar, adoption of technology, and historical epidemiology. The research also showed that such analysis could reveal the concepts of great societal interest across time and the associated linguistic changes (eg, the shifting use of “The Great War” and “World War I” to discuss the same event) [4]. Funk, in a frequency analysis of a corpus of journal articles exploring changes in the medical library profession over 50 years, revealed changing trends in the profession, such as the growing preference for evidence-based information and an increasing focus on health in addition to medicine [5].

To the best of our knowledge informed by a thorough literature search, no published quantitative studies document changes in HIV-related terminology over time. The few published works investigating HIV terminologies are qualitative and focus on word use in a specific context. For example, Mupenda et al interviewed people living with HIV in the Democratic Republic of the Congo about the labels applied to them in their community and social groups. Using text mining software to code and analyze the information, the study identified mostly derogatory labels (eg, “a disease that one chooses to get,” “a sick hen,” and “walking or standing corpse”) [6]. The study, however, did not explore how the labeling may have changed over time. Duby et al interviewed women in Africa who had previously participated in an HIV prevention study to better understand how participants interpret questions related to sexual behaviors. The study results highlighted how cultural biases, misunderstanding of research terms, and incorrect or absent translations for vernacular expressions can negatively affect HIV research [7]. Several nonresearch publications also make prescriptive recommendations about preferred terminology in clinical, community, and research contexts. For example, an annotation of terminology recommendations developed by advocates and activists living with HIV recommends using language that puts people first, avoiding the word “infection” unless necessary, and not merging HIV and AIDS into a single term [8].

### The Study

To expand the research on HIV terminology into a quantitative, temporal characterization of HIV terminology, a digitized dataset of abstracts presented at the International AIDS Conference (IAC) from 1989 to 2014 was analyzed. The IAC abstracts were used for this study because they were available from a sole source, the International AIDS Society. In addition, the IAC, initially convoked in 1985, represents the global response to HIV [9]. Analysis of the IAC dataset involved a hybrid process that integrated automated text mining and visualization with manual subject matter expert reviews. The text mining analysis incorporated algorithms tested successful in similar research to determine word relevancy and for term extraction [10,11]. Data processing for this study was conducted using the KNIME Analytics Platform, which is an open-source software used widely in the academic community for text mining [12]. The KNIME Analytics Platform includes modules for data blending, data and text mining, and machine learning, as well as other technical components. This study used the KNIME text mining module that is designed to perform statistical analysis on textual data (represented in a term-document frequency matrix) for further data analysis [13].

Results of the text mining analyses were visualized using Tableau Desktop to identify and highlight trends in HIV-related terminology use across the conference years. Tableau is a business intelligence interactive data visualization and dashboard tool [14]. These results can provide some insight and help generate hypotheses about the evolving historical and sociocultural context of the HIV epidemic.

### Study Objectives

The rationale for this study was multifold. First, we wanted to investigate using automated text analysis to identify terminology trends in a health domain, specifically HIV. We also aimed to construct a digitized corpus representing the evolution of HIV terminology in scientific literature. In addition to its value as a historical document, such a representation may provide a foundation for follow-up studies on the correspondence between social and linguistic changes, the uniformity of linguistic changes, and the implementation of terminology guidelines. It may also be useful to include the results of this study in guidelines and training materials to illustrate the relationship between language and stigmatization of individuals living with a disease.

Although the study was largely exploratory, results were expected to include terminology changes reflecting (1) Reduced stigmatization of individuals living with HIV, and (2) New strategies to treat HIV and reduce HIV transmission.

The following research questions were investigated in this study: (1) What changes in HIV-related language, if any, can be determined from IAC abstracts over 25 years?; (2) Are there any HIV-related terms that appear in abstracts only in specific years?; and (3) How does the HIV-related language used in the abstracts reflect the history of the HIV epidemic during the same 25-year period?

## Methods

### Project Workflow

**Figure 1:**
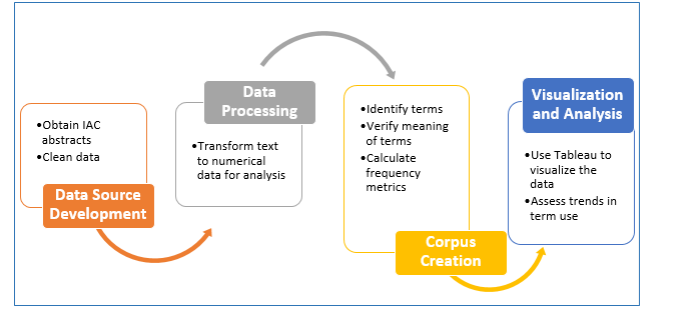
Project workflow. IAC: International AIDS Conference.

The study progressed in four stages as shown in Figure 1: data source development, data processing, terminology corpus creation, and visualization and analysis.

### Data Source Development

A total of 88,922 abstracts were obtained from the International AIDS Society for IAC conference years 1989 to 2014. The number of abstracts per IAC conference year varied, ranging from 5547 in 1989, 10,193 in 2006, to 2335 in 2014. The extensive dataset was delivered in comma-separated values files. The comma-separated values files were imported into an Excel (Microsoft) spreadsheet for data cleaning, a step necessary to convert the machine readable data into a more appropriate dataset for text mining. The data cleaning process included eliminating duplicate abstracts and removing HTML characters. This process did not change any words or alter the contents of the abstracts. The cleaned-up data were then imported into a Microsoft SQL Server relational database. The data from the Microsoft SQL Server database were converted to text files for data processing and text mining analysis.

### Data Processing

Using the KNIME software, the IAC abstracts were parsed to extract and represent the abstract text as a data structure. The parsed text was represented by the inherent KNIME data types called “DocumentCell” and “DocumentValue.” The parsed text was collected and saved as a data object or vector in which each element is an abstract. This processing allowed the application to manipulate the abstract text for further analysis, specifically to identify terms of interest and estimate relevant metrics of frequency. The KNIME software was then used to mine the partially processed data to create a terminology corpus of key HIV terms for analysis. The process began by preprocessing the data object of abstracts to filter out irrelevant terms (eg, stop words [the, a, an], numbers, punctuation, and diacritics [umlauts, accents]).

### Terminology Corpus Creation

The process to create the terminology corpus continued with the use of the KNIME Predictive Analytics software to separate the abstracts by conference year into unigrams. The number of words per abstract per conference year ranged from 36,000 to 60,000. Each word was associated with a KNIME-calculated metric of document frequency (the number of abstracts in which a term appears in a conference year).

The unigrams were exported to an Excel (Microsoft) spreadsheet and organized in ascending order of document frequency. A visual assessment of the words and their associated document frequencies showed that the majority of the terms had a document frequency of over 100. An HIV subject matter expert reviewed the spreadsheet to identify an initial set of unigrams (eg, “HIV,” “AIDS,” and “therapy”) with a document frequency of 100 or higher to investigate further. The subject matter expert also scanned the spreadsheet for any unigrams below the frequency threshold but judged relevant based on her HIV-related expertise. Given the scope of the project and the data import limitations of the free Tableau Desktop visualization software used, the subject matter expert limited this initial set of unigrams to 15 to 20 key unigrams. A second HIV subject matter expert reviewed the set of key unigrams to confirm their HIV-related relevancy.

KNIME Predictive Analytics software was again used to generate related bigrams (two-word terms) and trigrams (three-word terms) from the key unigrams. For example, the bigram “antiretroviral therapy” and the trigram “combination antiretroviral therapy” were generated from the unigram “therapy.” From the set of 15 to 20 key unigrams, the KNIME software generated approximately 15,000 bigrams and trigrams.

Again, using an initial document frequency threshold of 100, two HIV subject matter experts reviewed the unigrams, bigrams, and trigrams to identify key 1-, 2-, 3-, and 4-word terms to create the HIV terminology corpus. Both subject matter experts completed this step independently and then compared the results to ensure that all possible terms were included in the corpus. Drawing on their knowledge of HIV, the subject matter experts also included some unigrams, bigrams, and trigrams with a document frequency of less than 100 (eg, “drug holiday”) and some 4-grams (eg, “people who inject drugs” and “highly active antiretroviral therapy”) to the terminology corpus. The final set of HIV-related terms (the terminology corpus) included 1-, 2-, 3-, and 4-word terms.

The objective of this research was to evaluate the terms used in the IAC abstracts. Hence, synonyms were not normalized, and the words used in the abstracts were retained in the terminology corpus unchanged, except for the normalizations mentioned below. Research investigating use of Term Frequency-Inverse Document Frequency (TF-IDF) for query retrieval noted that the algorithm does not equate a word with its plural and that this limitation could potentially produce inaccuracies [10]. To overcome this limitation of the TF-IDF calculation, the terminology corpus creation process used for this study accounted for plural terms. In addition, terms with symbols were normalized by converting the symbols to equivalent terms. For example, *HIV+* was converted to “HIV positive.” Related terms, abbreviations, and acronyms for terms (eg, “ART” for “antiretroviral therapy”) were also added to the terminology corpus. Any acronym representing more than one word or phrase (eg, “STI,” the acronym for both “structured treatment interruption” and “sexually transmitted infection”) was not included in the terminology corpus. Once the terminology corpus was finalized, related terms and their abbreviations and acronyms were grouped into categories (eg, “HIV prevention” and “living with HIV”). The terms included in each category were mutually exclusive, and each term was classified into one category.

Given the extent of the IAC dataset (80,000 abstracts), it was impossible to verify that the meaning or contextual use of terms in the terminology corpus was consistent across all conference years. However, the validity of the terminology corpus was confirmed in a subset of abstracts (see Multimedia Appendix 1 for the final terminology corpus).

### Metrics Evaluated

Two frequency metrics were calculated to identify changes in HIV terminology use within and across conference years: relative term frequency (relTF) and TF-IDF. To derive these metrics, the following additional frequencies were calculated: absolute TF, TF, document frequency, IDF, and moving average of relTF.

#### Relative Term Frequency

RelTF is a normalized metric used to compare HIV terminology within and across conference years. To calculate relTF, absolute TF was first estimated by conference year. Absolute TF represents the total number of times that a term appears in all abstracts in a conference year. For example, if conference year 1 includes three abstracts and the term “AIDS patient” appears 5 times in abstract 1, 10 times in abstract 2, and 15 times in abstract 3, the absolute frequency for “AIDS patient” in year 1 is 30.

TF represents absolute TF normalized by the number of *all* words appearing in all abstracts in a conference year. The total number of *all* words is called *the vocabulary size*. TF is a normalized value used to compare HIV terminology use within and across conference years.

TF within a conference year was calculated as shown in equation 1, where *T* is a specific term, and *Y* is a specific conference year:

(1) *TF*(*T,Y*)=(*absolute term frequency*)/(*document corpus*)

Table 1 provides an example contrasting absolute term frequencies and term frequencies by conference year for two terms.

RelTF represents TF multiplied by 1,000,000. Thus, relative TF is the number of times in which a term is expected to appear in a document of 1,000,000 words.

**Table 1:** Absolute term frequency and term frequency for select terms.

Term	IAC^a^conference year	Vocabulary size	Absolute term frequency	Term frequency
Affected communities	1989	337,263	1	0.00000242
Affected communities	1990	213,980	2	0.00000775
Affected communities	1991	239,047	2	0.00000695
Affected communities	2010	653,819	29	0.00004110
Affected communities	2012	413,162	9	0.00002039
Affected communities	2014	269,633	10	0.00003399
AIDS carrier	1989	337,263	7	0.00001693
AIDS carrier	1990	213,980	1	0.00000388
AIDS carrier	1993	355,524	2	0.00000478
AIDS carrier	1994	240,841	4	0.00001424
AIDS carrier	1998	559,343	3	0.00000499
AIDS carrier	2002	845,289	2	0.00000218
AIDS carrier	2004	866,840	2	0.00000216

^a^IAC: International AIDS Conference.

### Term Frequency-Inverse Document Frequency

TF-IDF is a normalized metric that reflects the contextual importance of a term in abstracts for a specific conference year. To calculate TF-IDF, document frequency and IDF were calculated first. Abstract document frequency represents the number of abstracts in which a term appears in a conference year. At this level, each abstract in a conference year was considered independently. Document frequency at the abstract level is used to compare HIV terminology use within but not across conference years. Conference year document frequency represents the number of conference years in which a term appears in abstracts. Accordingly, all abstracts in a conference year including a term were combined and considered as a single document. Thus, at this level, document frequency ranged from 1 to 16, the number of conference years represented in the IAC dataset. The document frequencies by conference year were used as interim metrics to calculate IDF.

The frequency with which a term is used may not reflect its topical importance. For example, across the IAC abstracts, the conjunction “and” appears much more frequently than any HIV-related term. IDF gives more weight to key, less frequently used terms and less weight to less relevant, more frequently used terms. The intent is to reduce the absolute TF by a factor that grows with its TF. For this study, IDF was estimated by conference years.

IDF was calculated as shown in equation 2, where *T* is a specific term, *N* is the number of conference years, and *DF(T)* is the number of conference years that include abstracts in which a specific term appears:

(2) *IDF*(*T*)=log(1+*N*/(*DFT*)))

TF-IDF represents the product of TF (absolute TF normalized by the total number of words in all abstracts in a conference year) and IDF (the number of conference years that include abstracts in which the term appears).

TF-IDF for a conference year is calculated by multiplying TF by IDF as shown in equation 3, where absolute frequency *(T,Y)* is the number of times term T appears in conference year *Y*, vocabulary size *(Y)* is the number of terms that appear in abstracts in conference year Y*, N=16* is the number of conference years represented in the IAC dataset, and document frequency *(Y)* is the number of conference years in which term *T* appears in at least one abstract:

(3) *TFIDF*(*T,Y*)=TF*IDF=[(*absolute frequency *(*T,Y*))*/*(*vocabulary size document corpus *(*Y*))]*[*log*(*1+N/*(*document frequency*(*T*)))]

#### Moving Average of Relative Term Frequency

A moving average is used to *smooth out* random variations across time to make trends more evident. Moving averages are calculated for overlapping time spans rather than for discrete, mutually exclusive periods of time.

For this project, the moving average of relTF was estimated for overlapping conference years as shown in equation 4, where *t* is the conference year:

(4) *Mean Average*(*t*)=(*X*(*t-2*)+*X*(*t-1*)+*X*(t))/*3*

### Data Visualization

Once the terminology corpus of key HIV-related terms and their associated frequency metrics were calculated, Tableau was used to extract the data from the relational database and display the information in dashboards. The dashboard displays included line graphs, tables, and word clouds. A panel that included HIV public health subject matter experts, medical librarians, evaluation specialists, and technical experts in data analysis reviewed the dashboards to identify changes in HIV terminology across IAC conference years.

## Results

### Analysis by Terminology Category

The following results include both line graphs and word clouds and are grouped into two categories: people descriptors and treatment descriptors.

### People Descriptors

**Figure 2:**
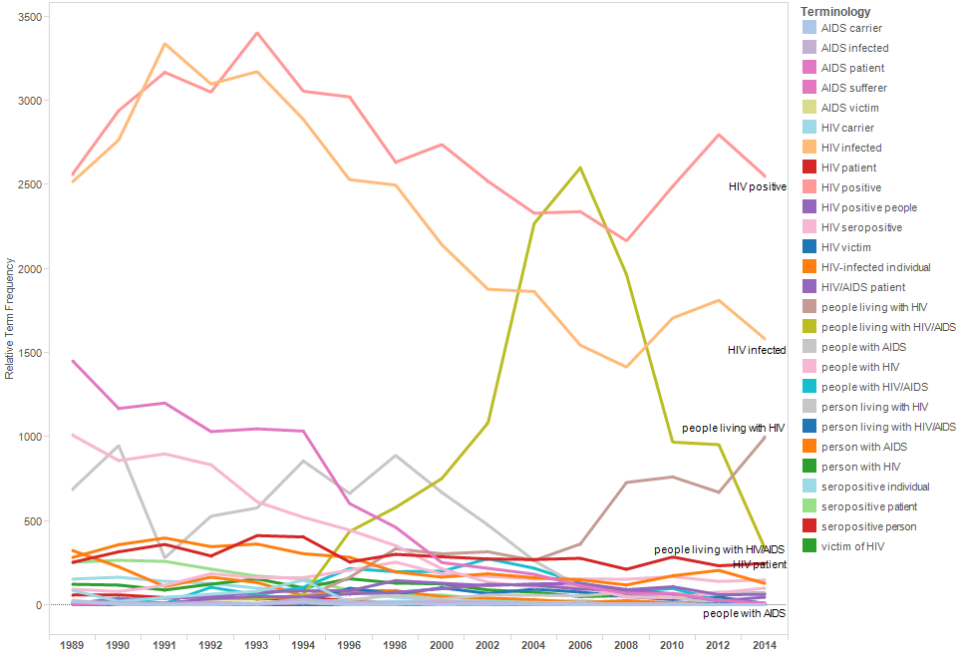
Relative term frequencies for terms associated with the category “living with HIV.”.

Figure 2 shows the relTFs for terms in the category “living with HIV.” Use of terms identifying individuals as “carriers” or “victims” of HIV (eg, “AIDS carrier,” “AIDS victim,” “HIV carrier,” and “HIV victim”) gradually disappeared from IAC abstracts, rarely appearing after 2008. Use of the terms “HIV positive” and “HIV infected” peaked in the early-1990s and then declined as evidenced by their decreasing relTFs.

**Figure 3:**
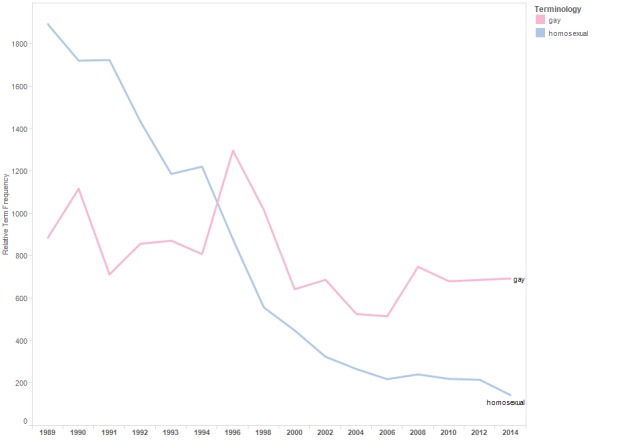
Relative term frequencies for terms associated with the category “homosexual.”.

Figure 3 is a line graph displaying the relTFs for the terms “gay” and “homosexual” included in the category “homosexual.” Use of both terms declined through conference year 2014.

**Figure 4:**
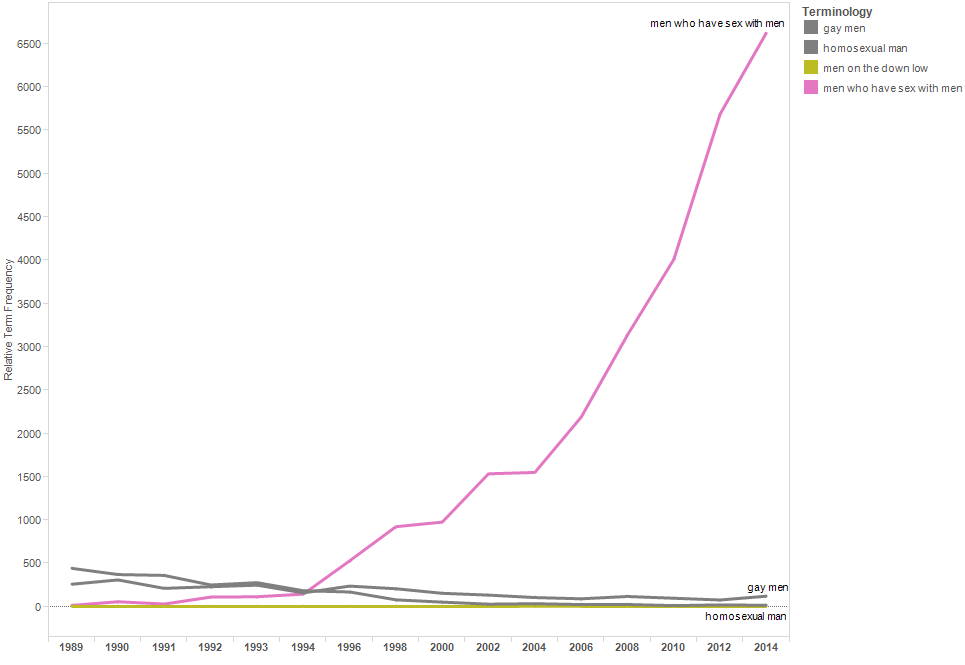
Relative term frequencies for terms associated with the category “MSM.”.

Figure 4 is a line graph showing the relTFs for the terms included in the terminology category “MSM.” The terms “men who have sex with men” and “MSM” were rarely used until 1994; subsequent use of these terms increased through 2014.

**Figure 5:**
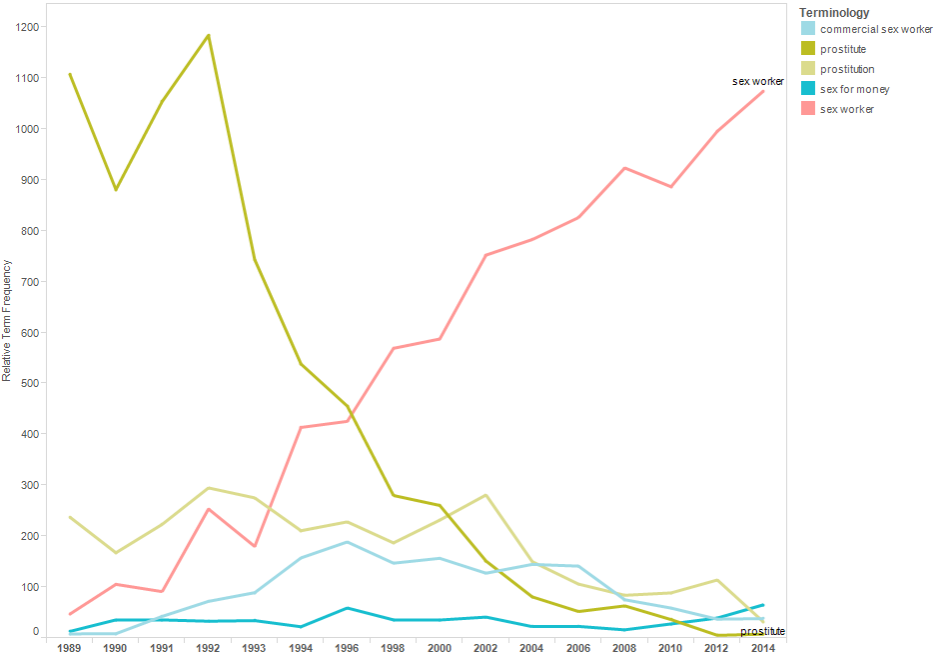
Relative term frequencies for terms associated with the category “sex worker.”.

Figure 5 shows a line graph of the relTfs for the terms included in the terminology category “sex worker.” Across the conference years, the most notable contrast is the declining use of the term “prostitute” and the increasing use of the term “sex worker.”

**Figure 6:**
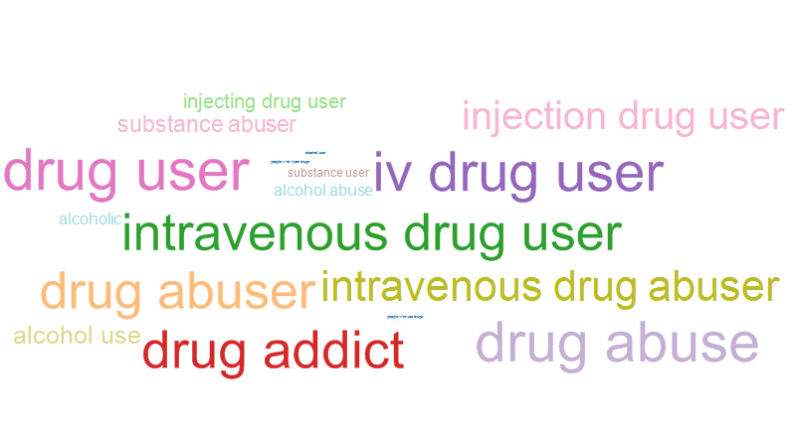
Word cloud for terms associated with the “alcohol and drug use” category in conference year 1989.

**Figure 7:**
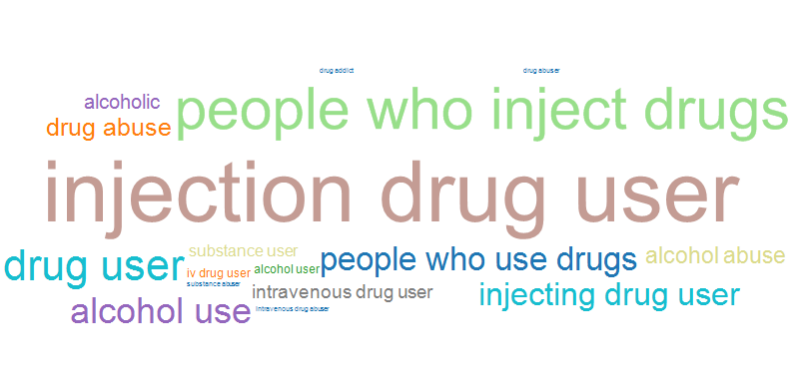
Word cloud for terms associated with the “alcohol and drug use” category in conference year 2014.

Figures 6 and 7 show word clouds representing the TF-IDFs for terms included in the category “alcohol and drug use” for conference years 1989 and 2014, respectively. Word clouds were used to highlight results of this category because of the dramatic shift noted between terms used in 1989 and 2014 that are more evident in the word cloud than a line graph. The word clouds illustrate that the use of the terms “drug addict,” “drug abuser,” and “drug abuse” in 1989 were replaced by the terms “people who use drugs” and “people who inject drugs” by 2014.

**Figure 8:**
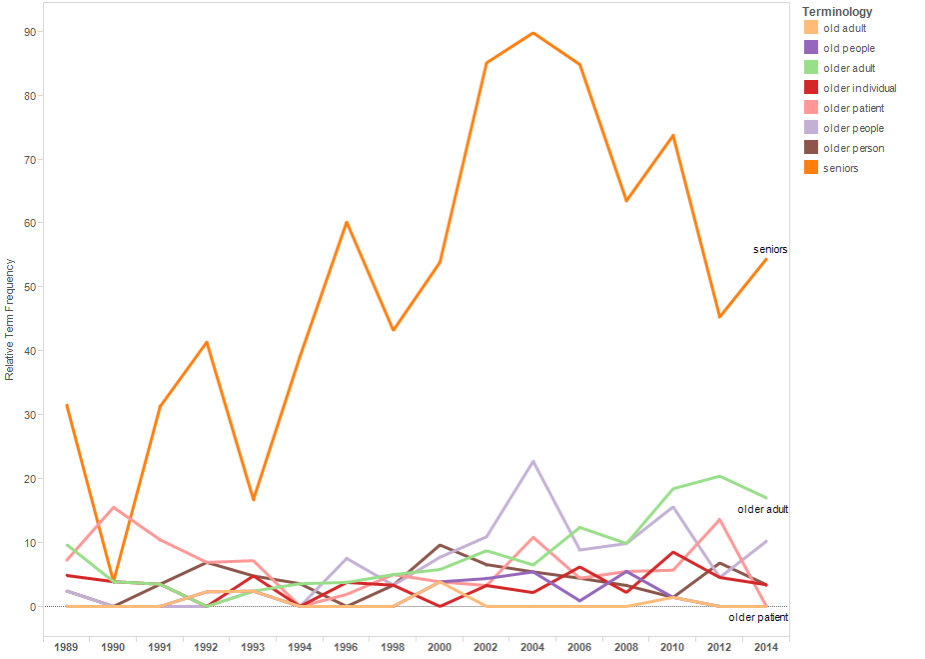
Relative term frequencies for terms associated with the “older adults” category.

Figure 8 shows a line graph of the relTFs for the terms included in the terminology category “older adults.” Starting in the early 1990s, the term “seniors” was used more frequently that the term “older patient.”

**Figure 9:**
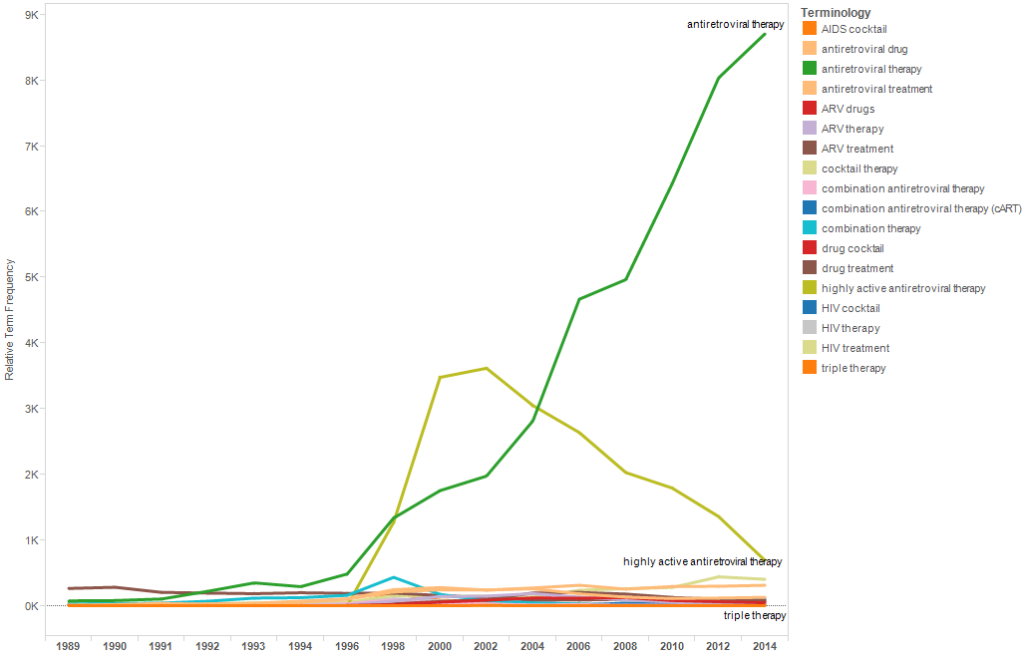
Relative term frequencies for terms associated with “antiretroviral therapy” category.

**Figure 10:**
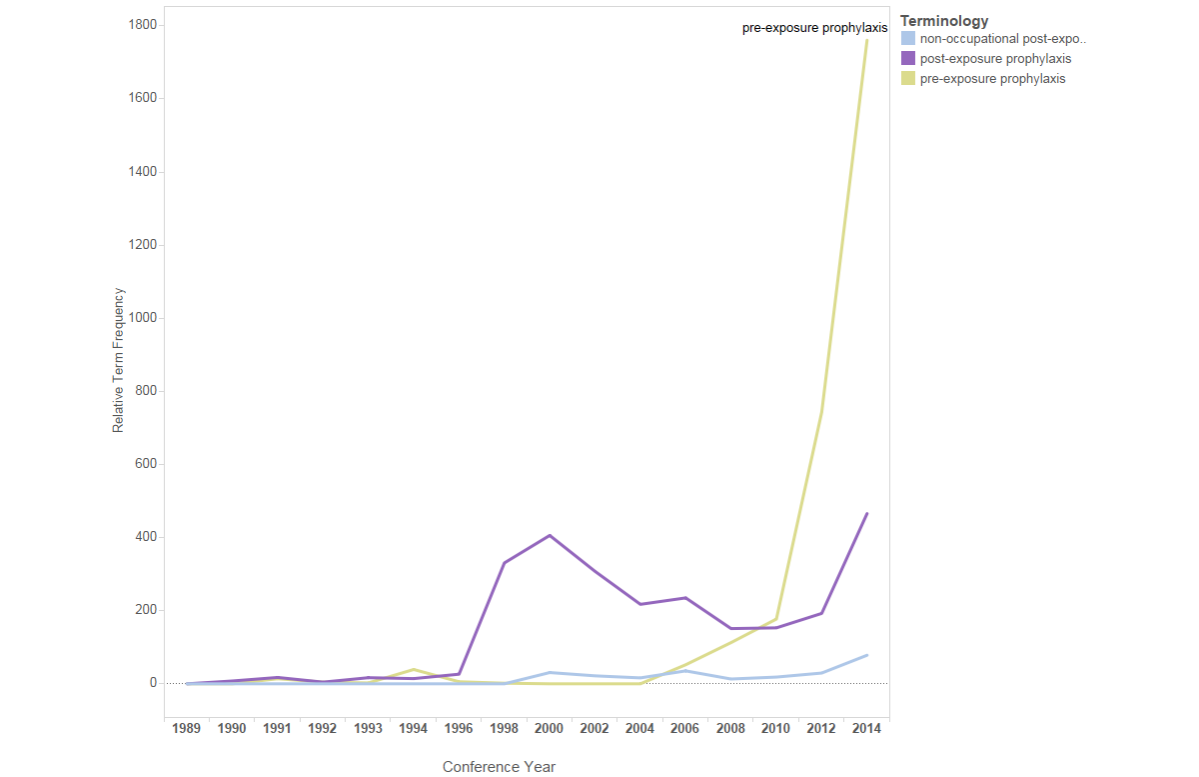
Relative term frequencies for terms associated with the “pre-exposure prophylaxis and post-exposure prophylaxis” category.

### Treatment Descriptors

Figure 9 shows a line graph with the relTFs for the terms in the category “antiretroviral therapy.” The relTF of the term “highly active antiretroviral therapy” increased from 1998 to 2002 and then decreased through 2014. The terms “AIDS cocktail” and “HIV cocktail” began appearing in abstracts in the late 1990s, appeared through the mid-2000s, and then were not used again until the late 2000s.

Figure 10 shows a line chart of the relTFs for the terms included in the terminology category “pre-exposure prophylaxis and post-exposure prophylaxis.” Pre-exposure prophylaxis and post-exposure prophylaxis refer to the use of antiretroviral drugs before and after exposure to HIV, respectively, to reduce the risk of HIV infection [15,16]. From 1996 to 2000, the relTF of “post-exposure prophylaxis” increased and then declined from 2000 to 2010. From 2010 to 2014, the relTF of “pre-exposure prophylaxis” and “treatment as prevention” increased.

#### Inception of Terms

Results of this research also include some terms that appeared in the IAC abstracts only for a limited number of conference years. Use of the term “AIDS epidemic” was dominant from 1989 to 1991 and then declined in use. In contrast, use of the term “HIV epidemic” increased through 2014. Beginning in the mid-1990s, the term “treatment experienced” appeared with increasing frequency in the abstracts.

Use of the term “treatment interruption” began to increase in 1997, appearing in IAC abstracts with increasing frequency until 2004. Similarly, use of the related terms “stopping treatment” and “structured treatment interruption” peaked in 2000 and 2004, respectively, and then precipitously declined in use through 2014. Finally, beginning in the mid-1990s, the terms “continuum of care” and “HIV care continuum” appeared with increasing frequency in the IAC abstracts.

## Discussion

### Principal Findings

As anticipated, changes in HIV language use across the IAC abstracts illuminated in the Tableau-generated dashboards parallel real-world advances in HIV research and medical practice. Importantly, the changes also highlight the declining stigmatization of both people living with HIV and those at increased risk of HIV exposure and areas where greater sensitivity in terminology would be beneficial.

#### People Descriptors

Discovery of the virus that causes AIDS in 1981 [1] spurred research to better understand the natural history of HIV. This research led to the development of HIV tests able to detect HIV along the disease continuum before the onset of AIDS. Accordingly, across the succession of IAC conferences, abstract authors increasingly distinguished between “HIV infection” and “AIDS” as indicated by the declining frequency trends lines for terms such as “AIDS epidemic,” “AIDS patient,” and “HIV/AIDS patient” and the increasing the trend lines for the terms “HIV epidemic,” and “people living with HIV.”

Results reflect progress to destigmatize HIV and to avoid stigmatizing people at risk of HIV exposure. For example, the trend lines for “drug addict” and “prostitute” declined, whereas those for “people who use drugs” and “sex worker” increased. Similarly, the use of “homosexual” and “gay” declined in abstracts across conference years, whereas the use of “men who have sex with men” increased. The increasing use of the term “living with HIV” likely reflects efforts to destigmatize HIV, as well as the transition of HIV from a fatal to chronic disease and attention to distinguish HIV infection from AIDS.

At the same time, the results of this research suggest the need for greater efforts to destigmatize HIV. It is notable that while people living with HIV prefer language that puts the person before the disease (ie, person-first language) [2,8], even in 2014, the two most frequently used status-referents in the abstracts are “HIV positive” and “HIV infected.” As emphasized in an annotation of HIV terminology recommendations, the word “infected” can be particularly stigmatizing [8] (although declining in use, “HIV infected” appears frequently in abstracts through 2014). Similarly, across conference years, the term “senior” (traditionally used to describe people aged 65 years or older) was used more often than “older adult.” A glossary recommending socially neutral language noted that objections to the term might increase as more baby boomers reach the age of 65 years [17].

Some generally positive terminological shifts to avoid stigmatizing people affected by HIV also merit further discussion. For example, growing use of “men who have sex with men” to describe a group disproportionately affected by HIV reduces stigma and expands the descriptor to include men who do not identify as “gay.” At the same time, in an article by Kaplan et al, using “men who have sex with men” as an HIV risk category excludes some identities (eg, trans feminine people, particularly those from non-Euro-Atlantic cultures) [18]. This categorization, in turn, may prevent some individuals whose gender and sexual identities do not fit binary notions from seeking HIV-related services and participating in HIV research.

#### Treatment Descriptors

Results highlighted advances in HIV treatment that transformed HIV from a fatal to a chronic disease. The first antiretroviral drug for the treatment of HIV was approved by the US Food and Drug Administration (FDA) in 1987 [19]. However, the effectiveness of HIV monotherapy was inhibited by the ability of HIV to mutate and become drug resistant. Consequently, researchers began to investigate combinations of antiretroviral drugs to more effectively suppress the virus. The research to find a multidrug alternative to HIV monotherapy is reflected in use of the terms “AIDS cocktail” and “HIV cocktail,” in the IAC abstracts from the late 1990s to the mid-2000s and again in the late 2000s. With the approval of an increasing number of antiretroviral drugs and a landmark clinical trial definitively demonstrating the benefits of triple drug therapy in 1997, the terms “highly active antiretroviral therapy” or “HAART” and “antiretroviral therapy” or “ART” increasingly appeared in IAC abstracts through 2014 [20]. The term “highly active antiretroviral therapy” appeared first in IAC abstracts in 1998, spiking in frequency of use in 2002, and then declined in use through 2014. In contrast, the term “antiretroviral therapy” appeared in IAC abstracts across conference years. As combination therapy progressed from an HIV treatment strategy under investigation to the universal standard of care, emphasis on its potent antiviral activity because superfluous. Accordingly, use of the abbreviated “antiretroviral therapy” surpassed “highly active antiretroviral therapy” by 2006 and continued to increase as the preferred term through 2014.

The increasing availability and use of HIV drugs is also reflected in the introduction of the term “treatment experienced” in IAC abstracts in 1996. Increasing use of the term through 2014 likely reflects the urgency to investigate treatment challenges facing the growing population of HIV-infected individuals on long-term antiretroviral therapy (ART).

The increasing use of the term “continuum of care” in IAC abstracts beginning in the mid-1990s also points to the transition of HIV to a chronic disease. The term reflects increasing focus on the span of HIV care from detection and diagnosis to long-term treatment with ART.

Changes in term use in the IAC abstracts also reflect definitive findings in HIV research. For example, in 2006, a major HIV study comparing continuous with episodic ART was halted when preliminary findings demonstrated that episodic ART increased the risk of HIV progression [21,22]. Not surprisingly, use of the term “structured treatment interruption” declined abruptly in conference year 2006. Another example is the spike in use of the term “pre-exposure prophylaxis” or “PrEP” in 2012, the year that FDA approved use of the HIV drug Truvada for pre-exposure prophylaxis [23].

### Terminology Recommendations

Results of this study demonstrate changes in HIV-related language reflective of historical changes in the HIV epidemic. Coupling results of this quantitative study with findings from related qualitative research [6-8], a number of terminology recommendations are provided as follows:

- Use consistent, biomedically accurate language to describe HIV. For example, use “HIV” and not “HIV/AIDS,” which equates the disease with its terminal stage.
- When describing people living with HIV, put the person before the disease (eg, use “person with HIV” and not “HIV-infected person”).
- Avoid language that stigmatizes people at increased risk of HIV infection. For example, use “drug user” not “drug abuser” and “sex worker” not “prostitute.”
- Consider group and personal preferences when communicating with and about people affected by HIV. For example, follow the example of the individual or group when using terms such as “gay” or “men who have sex with men.”
- Avoid language that assigns blame. For example, use “the treatment failed” not “the individual failed treatment.”

We conclude that adoption of these recommendations will further efforts to destigmatize HIV and, most importantly, facilitate effective communication between health professionals and people affected by HIV.

### Research Recommendations

The following are recommendations for future research aimed at evaluating changes in HIV-related terminology over time:

- Conduct a study evaluating results of these analyses on people affected by HIV, including people living with HIV, HIV clinicians, outreach workers, case managers, and HIV researchers to gain insight on real-world experience with HIV terminology, which will further evaluate the impact of HIV terminology on language perceptions and importance
- Import data from future IAC conference abstracts into Tableau Desktop, and program the system to automatically update the dashboards based on the new data.
- Employ a more robust version of Tableau Desktop to overcome the data import limits of the free version of the visualization software, and expand the HIV terminology corpus for analysis.
- Use the methods described for this project to analyze other datasets of HIV-related terms, and compare the results of the analyses.

Further research is also needed to identify optimal strategies to promote adoption of the terminology recommendations provided. Future studies may also investigate how implementation of the terminology recommendations enhances communication about HIV, especially between people affected by HIV and health care providers.

### Study Limitations

The HIV terminology corpus developed for analysis was based on unigrams of interest selected by HIV subject matter experts, an acknowledged limitation of this project. This nonautomated selection of key unigrams was dependent on the subject matter experts’ knowledge of HIV and their familiarity with trends in HIV terminology use. Automated term extraction is an efficient method to quickly extract terms from a large dataset; however, the method does not account for contextual differences. Although automated term extraction may have produced more terms for study analysis, manually term extraction allowed us to consider the contextual use of terms, which was especially important for terms with more than one meaning.

The study is limited to terminology used in IAC abstracts through conference year 2014. Although the 2016 IAC had taken place at the time of the study, the conference abstracts were not available for analysis.

This study was also restricted by the limited number of unigrams selected to build the HIV terminology corpus for analysis. The initial selection of unigrams was restricted by the data import limitations of the free Tableau visualization software used and the scope of the project. Expanding the choice of unigrams would have greatly extended the application processing and computational time required and resulted in an overwhelming number of terms from which to select a terminology corpus for analysis.

### Conclusions

Results of this study highlight changes in HIV terminology use over 25 years, including the addition, disappearance, and changing use of terms that reflect advances in HIV research and medical practice and efforts to use less stigmatizing language. Coupled with findings from related quantitative research, HIV-related terminology recommendations based on results of this study are included. Adoption of these recommendations will further efforts to use less stigmatizing language, improve retention in care, and facilitate effective communication between health care professionals, the media, and people affected by HIV.

## Appendix

Multimedia Appendix 1 HIV terminology corpus.

## Abbreviations

ART: antiretroviral therapy
FDA: Food and Drug Administration
IAC: International AIDS Conference
RelTF: relative term frequency
TF: term frequency
TF-IDF: term frequency-inverse document frequency
