# Supplementary material for: Trends in HIV Terminology: Text Mining and Data Visualization Assessment of International AIDS Conference Abstracts Over 25 Years
Source: JMIR Public Health Surveill. 2018 May 4;4(2):e50. doi: 10.2196/publichealth.8552 (PMC5960041; doi:10.2196/publichealth.8552)
Supplement: Multimedia Appendix 1 [file publichealth_v4i2e50_app1.pdf]

Appendix

HIV Terminology Corpus

Term	Category number	Category Name	Acronym available? (yes/no)	Acronym
AIDS carrier	1	Living with HIV	No	
AIDS infected	1	Living with HIV	No	
AIDS patient	1	Living with HIV	No	
AIDS sufferer	1	Living with HIV	No	
AIDS victim	1	Living with HIV	No	
HIV carrier	1	Living with HIV	No	
HIV infected	1	Living with HIV	No	
HIV patient	1	Living with HIV	No	
HIV positive	1	Living with HIV	No	
HIV positive people	1	Living with HIV	No	
HIV seropositive	1	Living with HIV	No	
HIV victim	1	Living with HIV	No	
HIV/AIDS patient	1	Living with HIV	No	
HIV-infected individual	1	Living with HIV	No	
people living with HIV	1	Living with HIV	Yes	PLWH
people living with HIV/AIDS	1	Living with HIV	Yes	PLWHA
people with AIDS	1	Living with HIV	Yes	PWA
people with HIV	1	Living with HIV	Yes	PWH
people with HIV/AIDS	1	Living with HIV	Yes	PWHA
person living with HIV	1	Living with HIV	No	
person living with HIV/AIDS	1	Living with HIV	No	
person with AIDS	1	Living with HIV	No	
person with HIV	1	Living with HIV	No	
seropositive individual	1	Living with HIV	No	
seropositive patient	1	Living with HIV	No	
seropositive person	1	Living with HIV	No	
victim of HIV	1	Living with HIV	No	
alcohol abuse	2	Alcohol and Drug Use	No	
alcohol use	2	Alcohol and Drug Use	No	
alcohol user	2	Alcohol and Drug Use	No	
alcoholic	2	Alcohol and Drug	No	

Term	Category number	Category Name	Acronym available? (yes/no)	Acronym
		Use		
drug abuse	2	Alcohol and Drug Use	No	
drug abuser	2	Alcohol and Drug Use	No	
drug addict	2	Alcohol and Drug Use	No	
drug user	2	Alcohol and Drug Use	No	
injecting drug user	2	Alcohol and Drug Use	No	
injection drug user	2	Alcohol and Drug Use	Yes	IDU
intravenous drug abuser	2	Alcohol and Drug Use	No	
intravenous drug user	2	Alcohol and Drug Use	No	
iv drug user	2	Alcohol and Drug Use	No	
people who inject drugs	2	Alcohol and Drug Use	No	
people who use drugs	2	Alcohol and Drug Use	No	
substance abuser	2	Alcohol and Drug Use	No	
substance user	2	Alcohol and Drug Use	No	
AIDS transmission	3	HIV Transmission	No	
HIV transmission	3	HIV Transmission	No	
HIV/AIDS transmission	3	HIV Transmission	No	
mother to child transmission	4	Mother-to-Child Transmission	No	
MTCT	4	Mother-to-Child Transmission	No	
perinatal transmission	4	Mother-to-Child Transmission	No	
vertical transmission	4	Mother-to-Child Transmission	No	

Term	Category number	Category Name	Acronym available? (yes/no)	Acronym
azidothymidine	5	Zidovudine (AZT)	No	
AZT	5	Zidovudine (AZT)	No	
azt therapy	5	Zidovudine (AZT)	No	
azt treatment	5	Zidovudine (AZT)	No	
Retrovir	5	Zidovudine (AZT)	No	
ZDV	5	Zidovudine (AZT)	No	
zidovudine	5	Zidovudine (AZT)	No	
zidovudine therapy	5	Zidovudine (AZT)	No	
zidovudine treatment	5	Zidovudine (AZT)	No	
AIDS cocktail	6	Antiretroviral Therapy (ART)	No	
antiretroviral drug	6	Antiretroviral Therapy (ART)	No	
antiretroviral therapy	6	Antiretroviral Therapy (ART)	Yes	ART
antiretroviral treatment	6	Antiretroviral Therapy (ART)	No	
ARV drugs	6	Antiretroviral Therapy (ART)	No	
ARV therapy	6	Antiretroviral Therapy (ART)	No	
ARV treatment	6	Antiretroviral Therapy (ART)	No	
cocktail therapy	6	Antiretroviral Therapy (ART)	No	
combination antiretroviral therapy	6	Antiretroviral Therapy (ART)	No	
combination antiretroviral therapy (cART)	6	Antiretroviral Therapy (ART)	No	
combination therapy	6	Antiretroviral Therapy (ART)	No	
drug cocktail	6	Antiretroviral Therapy (ART)	No	
drug treatment	6	Antiretroviral Therapy (ART)	No	
highly active antiretroviral therapy	6	Antiretroviral Therapy (ART)	Yes	HAART

Term	Category number	Category Name	Acronym available? (yes/no)	Acronym
HIV cocktail	6	Antiretroviral Therapy (ART)	No	
HIV therapy	6	Antiretroviral Therapy (ART)	No	
HIV treatment	6	Antiretroviral Therapy (ART)	No	
triple cocktail	6	Antiretroviral Therapy (ART)	No	
triple therapy	6	Antiretroviral Therapy (ART)	No	
discordant	7	Discordant	No	
discordant couple	7	Discordant	No	
HIV serodiscordant	7	Discordant	No	
HIV serodiscordant couple	7	Discordant	No	
mixed HIV status	7	Discordant	No	
mixed HIV status couple	7	Discordant	No	
mixed serostatus	7	Discordant	No	
mixed serostatus couple	7	Discordant	No	
antiretroviral adherence	9	Adherence	No	
antiretroviral therapy adherence	9	Adherence	No	
drug adherence	9	Adherence	No	
medication adherence	9	Adherence	No	
treatment adherence	9	Adherence	No	
treatment compliance	9	Adherence	No	
gay man	10	MSM	No	
gay men	10	MSM	No	
homosexual man	10	MSM	No	
homosexual men	10	MSM	No	
men on the down low	10	MSM	No	
men who have sex with men	10	MSM	Yes	MSM
gay	11	Homosexual	No	
homosexual	11	Homosexual	No	
AIDS epidemic	12	Epidemic vs. Pandemic	No	
AIDS pandemic	12	Epidemic vs. Pandemic	No	

Term	Category number	Category Name	Acronym available? (yes/no)	Acronym
HIV epidemic	12	Epidemic vs. Pandemic	No	
HIV pandemic	12	Epidemic vs. Pandemic	No	
HIV/AIDS epidemic	12	Epidemic vs. Pandemic	No	
HIV/AIDS pandemic	12	Epidemic vs. Pandemic	No	
scourge	12	Epidemic vs. Pandemic	No	
antiretroviral failure	13	Treatment Failure	No	
antiretroviral therapy failure	13	Treatment Failure	No	
ART failure	13	Treatment Failure	No	
ARV failure	13	Treatment Failure	No	
drug failure	13	Treatment Failure	No	
medication failure	13	Treatment Failure	No	
therapy failure	13	Treatment Failure	No	
treatment failure	13	Treatment Failure	No	
AIDS dementia complex	14	AIDS Dementia Complex	Yes	ADC
AIDS encephalopathy	14	AIDS Dementia Complex	No	
HIV associated dementia	14	AIDS Dementia Complex	No	
HIV encephalopathy	14	AIDS Dementia Complex	No	
AIDS defining condition	15	AIDS-Defining Condition	No	
AIDS defining disease	15	AIDS-Defining Condition	No	
AIDS defining illness	15	AIDS-Defining Condition	No	
commercial sex worker	16	Sex Worker	No	
prostitute	16	Sex Worker	No	
prostitution	16	Sex Worker	No	
sale of sexual services	16	Sex Worker	No	
sex for money	16	Sex Worker	No	
sex worker	16	Sex Worker	No	
healthy people	17	Living without HIV	No	
HIV negative	17	Living without HIV	No	
HIV seronegative	17	Living without HIV	No	

Term	Category number	Category Name	Acronym available? (yes/no)	Acronym
HIV uninfected person	17	Living without HIV	No	
normal people	17	Living without HIV	No	
seronegative individual	17	Living without HIV	No	
seronegative person	17	Living without HIV	No	
sexually transmitted disease	18	STI vs STD	Yes	STD
sexually transmitted infection	18	STI vs STD	No	
sexually transmitted infection (STI)	18	STI vs STD	No	
venereal disease	18	STI vs STD	Yes	
drug holiday	19	Treatment Interruption	No	
stopping therapy	19	Treatment Interruption	No	
stopping treatment	19	Treatment Interruption	No	
structured treatment interruption	19	Treatment Interruption	No	
therapy discontinuation	19	Treatment Interruption	No	
treatment interruption	19	Treatment Interruption	No	
antiretroviral naïve	20	Treatment Naïve	No	
antiretroviral therapy naïve	20	Treatment Naïve	No	
ART naïve	20	Treatment Naïve	No	
ARV naïve	20	Treatment Naïve	No	
drug naïve	20	Treatment Naïve	No	
treatment naïve	20	Treatment Naïve	No	
antiretroviral experienced	21	Treatment Experienced	No	
antiretroviral therapy experienced	21	Treatment Experienced	No	
ART experienced	21	Treatment Experienced	No	
ARV experienced	21	Treatment Experienced	No	
drug experienced	21	Experienced	No	

Term	Category number	Category Name	Acronym available? (yes/no)	Acronym
treatment experienced	21	Treatment Experienced	No	
AIDS test	22	HIV Test	No	
HIV test	22	HIV Test	No	
AIDS battle	23	Response to HIV	No	
AIDS response	23	Response to HIV	No	
battle against AIDS	23	Response to HIV	No	
battle against HIV	23	Response to HIV	No	
fight against AIDS	23	Response to HIV	No	
fight against HIV	23	Response to HIV	No	
HIV battle	23	Response to HIV	No	
response to AIDS	23	Response to HIV	No	
response to HIV	23	Response to HIV	No	
war against AIDS	23	Response to HIV	No	
war against HIV	23	Response to HIV	No	
affected communities	24	High Risk Groups	No	
affected community	24	High Risk Groups	No	
at risk population	24	High Risk Groups	No	
groups at risk	24	High Risk Groups	No	
high risk group	24	High Risk Groups	No	
client	25	Client vs. Patient	No	
patient	25	Client vs. Patient	No	
old adult	26	Older Adults	No	
old people	26	Older Adults	No	
older adult	26	Older Adults	No	
older individual	26	Older Adults	No	
older patient	26	Older Adults	No	
older people	26	Older Adults	No	
older person	26	Older Adults	No	
seniors	26	Older Adults	No	
continuum of care	27	Continuum of Care	No	
HIV care continuum	27	Continuum of Care	No	
HIV treatment cascade	27	Continuum of Care	No	
fixed dose combination	28	Fixed-Dose Combination	Yes	FDC
HIV prevention	29	HIV Prevention	No	
HIV/AIDS	30	HIV/AIDS	No	
non-occupational post-exposure prophylaxis	31	PrEP and PEP	Yes	nPEP
post-exposure	31	PrEP and PEP	Yes	PEP

Term	Category number	Category Name	Acronym available? (yes/no)	Acronym
prophylaxis				
pre-exposure prophylaxis	31	PrEP and PEP	Yes	PrEP
treatment as prevention	32	Treatment as Prevention	Yes	TaSP
